# Ergot in Menorrhagia

**Published:** 1857-09

**Authors:** 


*Ergot in Menorrhagia.*—Dr. J. McF. Gaston, in an article in the New Hampshire Journal of Medicine, recommends ergot as the most efficient remedy in menorrhagia. He combines with it iron, opium, or valerian, as circumstances require.

